# Impact of adapted taekwondo vs. multicomponent training on health status in independent older women: a randomized controlled trial

**DOI:** 10.3389/fpubh.2023.1236402

**Published:** 2023-10-10

**Authors:** Pablo Valdés-Badilla, Eduardo Guzmán-Muñoz, Tomás Herrera-Valenzuela, Braulio Henrique Magnani Branco, Jordan Hernandez-Martinez, Hadi Nobari

**Affiliations:** ^1^Department of Physical Activity Sciences, Faculty of Education Sciences, Universidad Católica del Maule, Talca, Chile; ^2^Sports Coach Career, School of Education, Universidad Viña del Mar, Viña del Mar, Chile; ^3^School of Kinesiology, Faculty of Health, Universidad Santo Tomás, Talca, Chile; ^4^School of Kinesiology, Faculty of Health Sciences, Universidad Autónoma de Chile, Talca, Chile; ^5^Department of Physical Activity, Sports and Health Sciences, Faculty of Medical Sciences, Universidad de Santiago de Chile (USACH), Santiago, Chile; ^6^Postgraduate Program in Health Promotion, Cesumar University, Maringá, Paraná, Brazil; ^7^Programa de Investigación en Deporte, Sociedad y Buen Vivir, Universidad de los Lagos, Osorno, Chile; ^8^Department of Physical Activity Sciences, Universidad de Los Lagos, Osorno, Chile; ^9^Faculty of Sport Sciences, University of Extremadura, Cáceres, Spain

**Keywords:** combat sports, resistance training, exercise, older adults, healthy aging, aging

## Abstract

This study, called the TKD and Aging Project, aimed to analyze and compare the effects of an adapted taekwondo program concerning multicomponent training on blood pressure, morphological variables, food consumption frequency, health-related quality of life (HRQoL), physical fitness, handgrip strength, and postural balance in independent older women. A randomized controlled trial study was conducted with parallel groups for 8 weeks (24 sessions of 60 min each), employing a double-blind design and incorporating repeated measures. Twenty-eight older women initially participated in the intervention. Three participants were excluded because they did not participate in the re-assessments. Thus, 14 older women from the adapted taekwondo group (TKD; age: 62.86 ± 2.38 years) and 11 from the multicomponent training group (MCT; age: 63.18 ± 1.94 years) participated in the final analysis. A two-factor mixed analysis of variance (ANOVA) model with repeated measures was performed to measure the time × group effect. The TKD showed significant improvements in the mental health (*p* = 0.024; ES = 0.91) and general health (*p* < 0.001; ES = 0.75) dimensions of the HRQoL, as well as in the chair stand (*p* = 0.001; ES = 1.18), arm curl (*p* < 0.001; ES = 2.10), 2-min step (*p* < 0.001; ES = 1.73), and chair sit-and-reach (*p* = 0.001; ES = 0.91) tests. Additionally, it showed a significant reduction in postural balance for the eyes-closed condition in the center of the pressure area (*p* = 0.021; ES = 0.89), mean velocity (*p* = 0.004; ES = 0.79), and mediolateral velocity (*p* < 0.001; ES = 1.26). However, the MCT showed significant increases in the general health (*p* = 0.013; ES = 0.95) dimension of the HRQoL and a significant reduction (*p* = 0.039; ES = 0.28) in the mediolateral velocity of postural balance for the eyes-closed condition. Multiple comparisons showed that the TKD scored significantly higher in the chair stand (*p* = 0.017; ES = 1.79), arm curl (*p* = 0.003; ES = 1.77), and 2-min step (*p* = 0.018; ES = 0.91) tests than the MCT. Compared to multicomponent training, taekwondo improves postural balance and provides better benefits in terms of physical fitness and HRQoL for older women. Therefore, it is possible to recommend it as a safe physical activity strategy, as long as it is well-dosed, since it showed high adherence to intervention in older women.

## 1. Introduction

Combat sports are considered risky physical activity due to their high injury rates in elite athletes (1, 2). For example, in Olympic combat sports athletes, the most frequent injuries and illnesses were 45.8% head/face injuries and bruises in boxing, 10.9% low back injuries in judo, 22.8% finger sprains in taekwondo, and 24.8% knee sprains in wrestling (1). Despite the aforementioned risks, combat sports are a prevalent physical activity practice (3). In addition, it has been suggested that, with appropriate dosage (e.g., selection of technical foundations, number of sessions and time, volume, intensity, and density), they can be used to train adults (4) and older people (5, 6), achieving benefits similar to other physical activity strategies at the physical, physiological, and psychoemotional levels. Recent systematic reviews (7, 8) have not been able to establish conclusive results regarding the favorable effects of Olympic combat sports on the health status of older people. However, the individual results of the studies analyzed indicate a significant reduction in fall risk (8) and an improvement in health-related quality of life (HRQoL) (7), with a mean adherence that is more significant than 80%.

In particular, adapted taekwondo interventions for older women have reported a significant increase in the number of repetitions of the chair stand (9, 10) and arm curl tests (10), as well as higher performance in the handgrip strength (HGS) (9, 11), and chair sit-and-reach tests (10). In addition, a significant reduction in systolic and diastolic blood pressure (9), a decrease in the seconds in the timed up-and-go (TUG) test (9, 10), significant improvements in brain-derived neurotrophic factor (10), and a substantial decrease in resting epinephrine (11) have also been reported to improve the HRQoL in healthy older women (10), hypertensive older women (11), and those with depression (9).

However, multicomponent training, which involves at least three essential physical qualities or abilities, typically resistance, aerobic capacity, balance, and flexibility (12, 13), has broad support and diffusion as a safe and effective physical activity intervention strategy for older people (12). Among the main benefits reported in an umbrella review of systematic reviews (12) were significant improvements in muscle strength, mobility, gait, balance, and general physical performance in community-dwelling frail older people. However, the review mentioned above indicated that there is still uncertainty about the most appropriate physical activity characteristics (type, frequency, intensity, duration, and combinations) for achieving the most beneficial and sustainable results over the long term (12).

Considering all of the above, it seems that both adapted taekwondo and multicomponent training achieve similar results concerning the general health status of older people (4, 6, 8, 12). Additionally, in Chile, older women are more sedentary, and the prevalence of being overweight/obese is higher among them than among men (14). According to the data available, this negatively affects the health status of Chilean older women (15), thereby justifying the promotion of novel physical activity strategies to encourage regular practice in this population group. In this sense, the present study aimed to analyze and compare the effects of an adapted taekwondo program concerning multicomponent training on blood pressure, morphological variables, food consumption frequency, HRQoL, physical fitness, HGS, and postural balance in independent older women. Based on previous systematic reviews (6–8, 12, 16), we hypothesized that adapted taekwondo would produce significantly greater effects on HRQoL and postural balance than a multicomponent training.

## 2. Materials and methods

### 2.1. Study design

**Figure 1:**
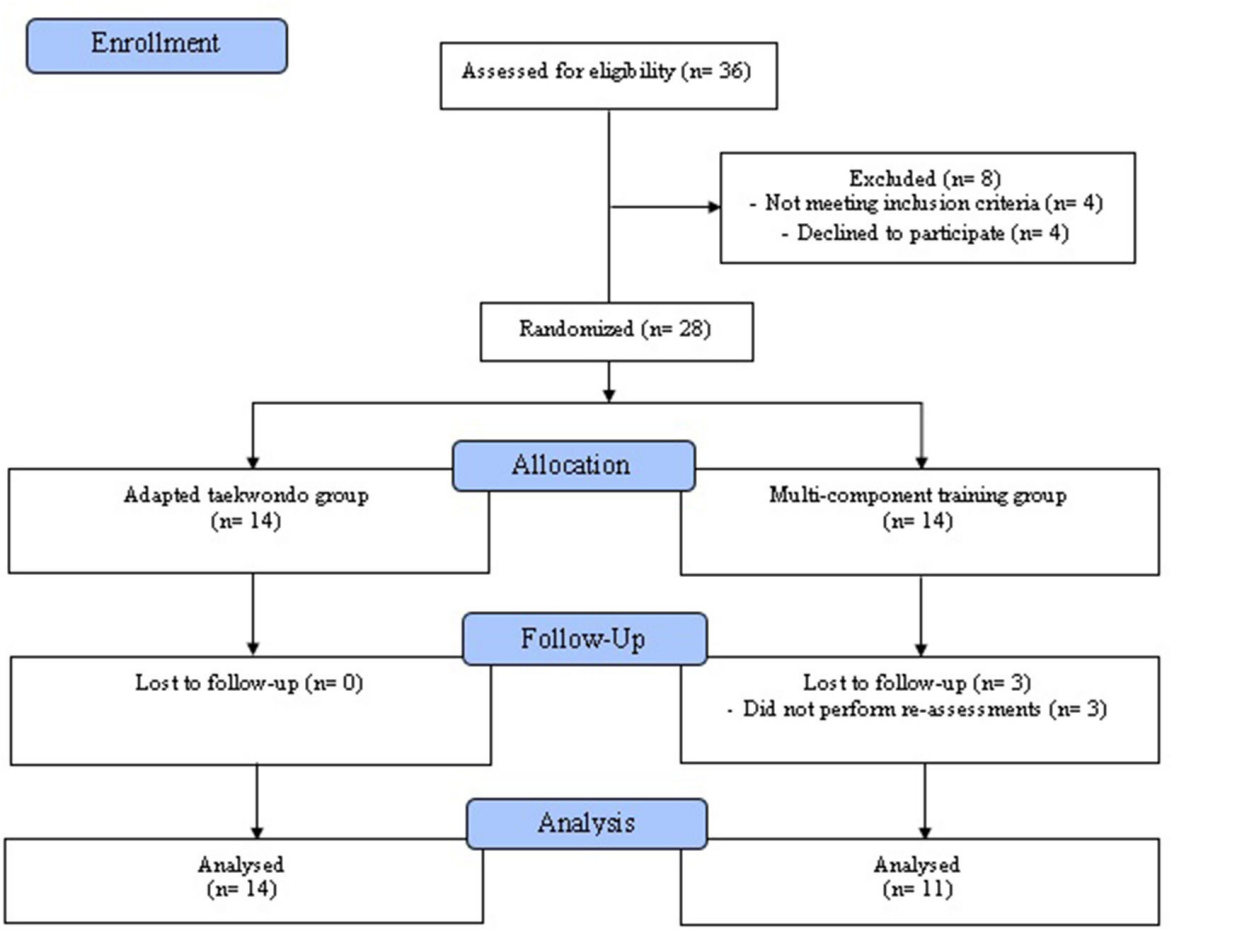
Study flowchart of the enrolment process, allocation, follow-up, and analysis of independent older women.

This study design included a randomized controlled trial with parallel groups (adapted taekwondo group: TKD; multicomponent training group: MCT), repeated measures, and double-blinding of both participants and assessors. A research randomizer website (https://www.randomizer.org) was used for randomization. The CONSORT guidelines (17) and a study protocol from the TKD and Aging Project (18) were used as the methodology. In addition, the study was registered in the Clinical Trial Protocol Registry and Results System (ClinicalTrials.gov) of the United States of America (Code: NCT05275140; http://clinicaltrials.gov/search?cond=NCT05275140, accessed on July 14, 2023). The interventions were conducted over the course of 8 weeks, comprising a total of 24 sessions. These sessions occurred three times a week, lasting 60 min each, specifically on Mondays, Wednesdays, and Fridays. Blood pressure, morphological variables, food consumption frequency, HRQoL, physical fitness, HGS, and postural balance were all assessed. All measurements were taken in the afternoon—between 14:00 and 15:00 h—and in the same location (sports center), with the control of variables, including temperature, and the researchers who conducted the pre- and post-assessments. The older women had no musculoskeletal and/or cardiorespiratory injuries during the intervention, and they exhibited no pain prior to the assessments or during the training sessions. The summarized inclusion criteria are described in Figure 1.

### 2.2. Participants

Twenty-eight older women initially participated in the intervention. The sample size calculation indicated that the ideal number of participants per group is 16. Based on previous studies (19), for this calculation, an average difference of 3.46 repetitions (chair stand test) was used as the minimum difference required for substantial clinical relevance, with a standard deviation of 3.38 repetitions, considering an alpha level of 0.05 with 90% power and an expected loss of 15%. GPower software (Version 3.1.9.6, Franz Faul, Universiät Kiel, Kiel, Germany) was used to calculate the statistical power. The inclusion criteria were as follows: (*i*) older women aged between 60 and 65 years; (*ii*) those presenting the ability to understand and follow instructions in a contextualized manner through simple commands; (*iii*) those who were independent, which was defined as having a score of at least 43 points on the Preventive Medicine Exam for the Older People of the Chilean Ministry of Health (20); and (*iv*) those with the ability to adhere to the requirement of at least 85% attendance at the scheduled sessions for intervention. Regarding the exclusion criteria, the following were considered: (*i*) having any disability condition; (*ii*) having musculoskeletal injuries or being treated for physical rehabilitation that prohibits them from doing their usual physical activities; and (*iii*) being unable to engage in physical activity either permanently or temporarily. Participants who met the inclusion criteria additionally had to attend all assessment sessions and finish at least 85% of the training sessions to be included in the final analyses. Three out of 28 older women considered for inclusion in the study were excluded because they did not participate in the re-assessments. Thus, 14 older women in the TKD (bipedal height: 1.55 ± 0.04 m) and 11 in the MCT (bipedal height: 1.54 ± 0.06 m) were analyzed.

All participants accepted the inclusion criteria for the usage and handling of the data by signing an informed consent form authorizing the use of the information for scientific purposes. The protocol was approved by the scientific ethics committee of the Universidad Católica del Maule, Chile (Number: N°29-2022) and was developed following the Declaration of Helsinki.

### 2.3. Primary outcomes

#### 2.3.1. Blood pressure

An automatic pressure monitor (08A, CONTEC, Germany) was purchased to measure the systolic and diastolic blood pressure. Following bladder emptying, the older women were assessed after at least 10 min of prior rest in a seated position with the back, arms, and legs uncrossed. The first assessment was taken in both arms to identify the arm with the highest blood pressure. The arm with the highest blood pressure (typically the dominant arm) was then subjected to two assessments, and a third evaluation was also conducted if the difference between the results was >5 mmHg. Procedures to measure blood pressure were carried out as proposed by Reddy et al. (21).

#### 2.3.2. Morphological variables

A digital scale (Seca 769, Germany; accuracy of 0.1 kg) was used for measuring body weight, and a stadiometer (Seca 220, Germany; accuracy of 0.1 cm) was used for measuring bipedal height. In accordance with the International Society for the Advancement of Kinanthropometry (ISAK) guidelines, all assessments were carried out by a level-II anthropometrist certified by the ISAK (22). The body mass index (BMI) of each older woman was also calculated by dividing weight in kilograms by the square of bipedal height in meters. To calculate the proportion of fat mass and fat-free mass, an eight-electrode tetrapolar bioimpedance device (InBody 570^®^, Body Composition Analyzers, Seoul, Korea) was used.

#### 2.3.3. Food consumption frequency

A modified version of an eating habits survey for older people was used to measure the food consumption frequency; this survey was validated by employing the Delphi method based on the opinions of 25 nutrition experts (23). Furthermore, the survey was designed with two self-application areas. (*i*) The first area comprises 12 questions with a minimum score of 1 and a maximum score of 5 (Likert scale), reflecting the frequency of healthy foods, including that of advised food groups. The scale ranges from not consuming (1 point) to the recommended day/week servings (5 points), with a score of the responses ranging from 12 to 60 points (a higher value indicates better eating habits) (23). (*ii*) The second area comprises 7 items related to unhealthy foods or food groups identified as promoters of chronic non-communicable diseases (sugary drinks, alcohol, fried foods, fast food, sweet snacks, and coffee). Six of the questions have the same score as that of the previous one (1, does not consume, to 5, over three servings per day/week), and one is rated from 1 to 3 (salt), reaching a value ranging from 7 to 33 points (higher values indicate unhealthy food choices). Bad eating habit, such as adding salt to meals without tasting them, was added (23).

#### 2.3.4. Health-related quality of life

This questionnaire was obtained using the Health Survey Short Form (SF-36) Version 2. It measures the attributes of eight health dimensions (24): physical function, physical role, body pain, general health, vitality, social function, emotional role, and mental health. Questions of each dimension are added together to form a scale, with the poorest health status for that dimension at 0 and the best health status at 100 (24).

#### 2.3.5. Physical fitness

Physical fitness was evaluated by conducting the Senior Fitness Test, which offers an assessment of outstanding reliability and simple application (25). The chair stand test was conducted as the first assessment in the battery to determine the lower limbs' muscle strength by counting how many repetitions were completed in 30 s. The arm curl test measured the upper limbs' muscle strength by counting the number of repetitions completed in 30 s while holding a 3-lb dumbbell. A 2-min step test was conducted to measure cardiorespiratory fitness by counting the number of knee raises each participant accomplished, reaching at least a 70-degree angle at their hip joint. The lower limbs' degree of flexibility was measured in centimeters during the chair sit-and-reach test. The back scratch test was conducted to measure the upper limbs' degree of flexibility in centimeters. The TUG test was conducted to measure agility and dynamic balance by encircling a cone at a distance of 8 ft (2.44 m) while controlling time in seconds.

#### 2.3.6. Handgrip strength

Using a hydraulic dynamometer (Camry, model EH101, China), HGS was measured in accordance with earlier recommendations (26). Older women were seated with their shoulders abducted, elbows flexed at 90° to one side of their bodies, forearms aligned neutrally, and wrists kept in a neutral position. The size of the hand was considered when adjusting the dynamometer, allowing for a functional and comfortable grip on the instrument with an adequate closure of the interphalangeal and metacarpal joints in the position of the fist, favoring contact between the first phalanges of the index and thumb. For each hand, three trials were made using the highest value possible in the three registers.

#### 2.3.7. Postural balance

Using a force platform (ArtOficio Ltd., Valparaíso, Chile), the center of the displacement of pressure was measured in accordance with earlier recommendations (27). The data were acquired with a sampling rate of 40 Hz. Postural balance was assessed both with the eyes open and closed, and each assessment lasted 30 s. The older women were instructed to remain as still as possible in the bipedal position, with their arms at their sides and their feet aligned approximately shoulder-width apart. Using the Matlab r2012a program (Mathworks Inc., Natick, USA), the area and velocity variables of the center of pressure were calculated.

### 2.4. Secondary outcomes

Baseline assessments of age (years), academic level (primary, secondary, bachelor, or postgraduate), civil status (married, separated, widowed, single, or others), and smoking status (yes or no) were made.

### 2.5. Intervention

The study protocol explained the TKD and MCT programs in detail (18). The general structure of the programs included a 10-min warm-up comprising joint mobility and low-intensity aerobic exercises, followed by a 40-min central part (TKD or MCT) and concluding with a 10-min cooldown through dynamic and static flexibility exercises over 8 weeks (24 sessions). A summary of the intervention dosage is presented in Table 1. The Polar Team app version 1.3 (Polar Electro Oy, Kempele, Finland) was used to continuously monitor the older women. The intensity of the interventions remained moderate to vigorous, with each older woman's maximum heart rate (HRmax) used as a control (between 50 and 70% of HRmax). This was carried out with a heart rate sensor strap (H10, Polar Electro Oy, Kempele, Finland), which was live-transmitted via bluetooth to a tablet (iPad 4, Apple, Inc., Cupertino, CA, USA). The sessions were led by master's degree students in physical activity sciences, who have worked with older people, and a National Sports Federation of Taekwondo WT-certified taekwondo instructor (for TKD).

**Table 1:** Intervention dosage.

**Program**	**Weeks**	**Fr (weekly)**	**TPS (min)**	**PE**	**Set**	**Rep**	**Rest**	**Intensity**
TKD	1–4	3	60	ULs	3	8	2-min	50–70% HRmax
				LLs				
				Forms	—	6		
	5–8			ULs	4	8		
				LLs				
				Forms	—	6		
MCT	1–4		60	RT	3	10	2-min	OMNI-RES (5–8 points)
				CF				50–70% HRmax
				APC				
	5–8			RT	4	10		OMNI-RES (5–8 points)
				CF				50–70% HRmax
				APC				

APC, Agility and postural control; CF, Cardiorespiratory fitness; Fr, Frequency; HRmax, Maximum heart rate; LL, Lower limbs; MCT, Multicomponent training group; OMNI-RES, OMNI-Resistance Exercise Scale of perceived exertion; PE, Physical exercise; Rep., Repetitions; RT, Resistance training; TKD, Adapted taekwondo group; TPS, Total time per session; ULs, Upper limbs.

The central part of the TKD program comprised non-contact exercises, which were broken up into 10 min of basic postures and specific technical foundations for the upper limbs (strikes and blocks) and 20 min of technical foundations for the lower limbs (displacement, postures, and kicks). These exercises were performed individually and in pairs, with and without the use of taekwondo implements (impact pads and shields). In addition, participants practiced these modality-specific choreographies or poomsae for 10 min. The amount of training was measured in sets and repetitions of the specific technical foundations, with a 2-min rest in between sets. Perceived exertion was measured using the Borg scale, which has a maximum rating value of 10 points (28).

**Figure 2:**
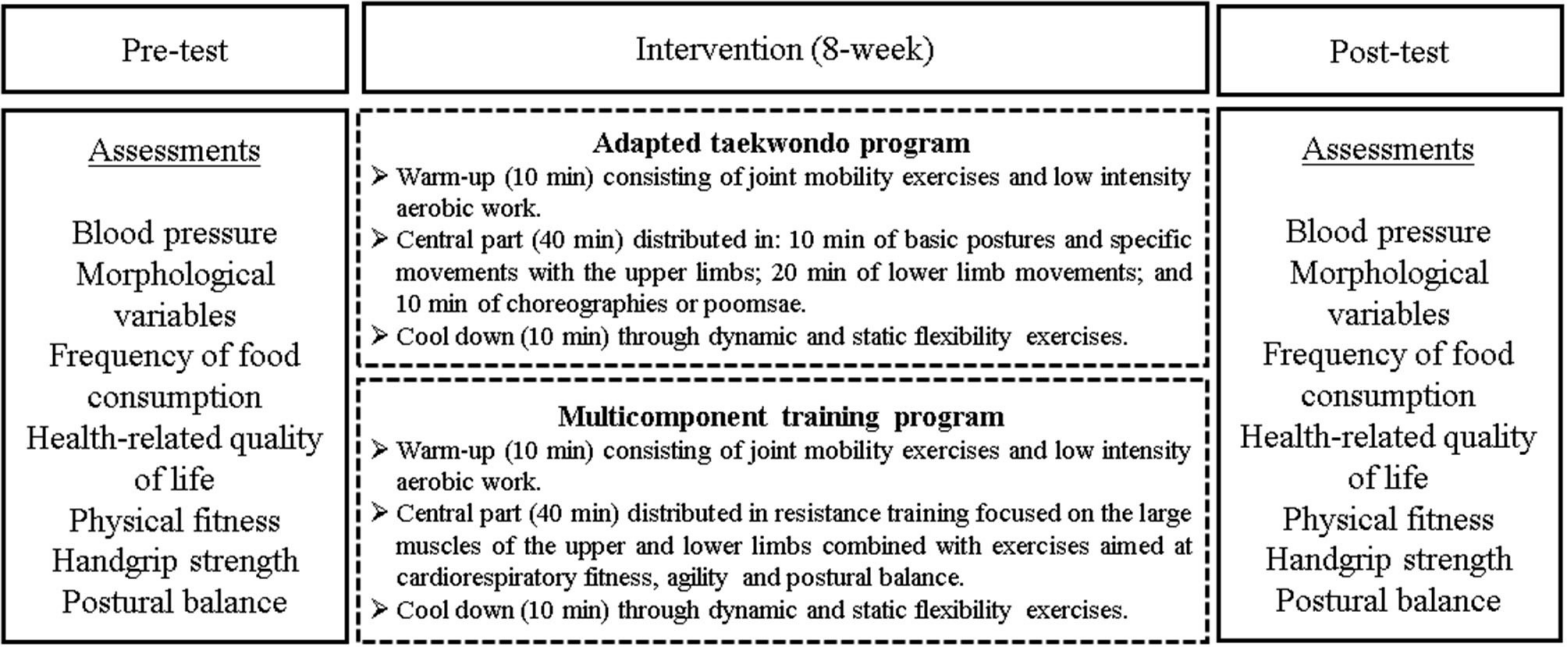
Assessments and regular sessions of the intervention.

The central part of the MCT program was a 40-min circuit of distributed work that included exercises for cardiorespiratory fitness, agility, and postural balance using elastic bands, poles, 2-kg medicine balls, and chairs. The exercises targeted the biceps, triceps, deltoids, latissimus dorsi, quadriceps, hamstrings, glutes, and gastrocnemius, which correspond to the large muscles of the upper and lower limbs. The first training volume (the first 4 weeks) consisted of three sets of 10 repetitions of each muscle activity, with a 2-min rest in between sets. Slow movements, lasting 2 s for concentric and 4 s for eccentric contractions, were used. The volume was increased to four sets of 10 repetitions of each muscular exercise with a 2-min rest in between sets (between weeks 5 and 8). The OMNI-Resistance Exercise Scale of perceived exertion was used to control the resistance training intensity, which ranged from moderate to vigorous (5–8 points) (29). The assessments and regular intervention sessions are summarized in Figure 2.

### 2.6. Statistical analysis

The analysis was conducted using the statistical program GraphPad Prism 9 (GraphPad Software, Inc., La Jolla, CA, USA). The mean and standard deviations were used to present the data. The analyzed outcomes complied with the normality of data through the Shapiro–Wilk test. Subsequently, a two-factor mixed analysis of variance (ANOVA) model with repeated measures was employed to measure the time × group effect of all the variables. A Bonferroni multiple comparison test (*post-hoc*) was conducted to determine the intra-group (pre vs. post) and inter-group (TKD vs. MCT) differences when the time × group interaction was significant. To determine the effect size of the time × group interaction, the partial eta square (ηp^2^) was calculated, which was interpreted considering the ηp^2^ values of 0.01, 0.06, and 0.14, which correspond to small, moderate, and large effect sizes (ES), respectively (30). For multiple comparisons, the ES was calculated using Cohen's d, considering a small (≥0.2), moderate (≥0.5), or large (≥0.8) effect (31) size. A significant difference was established for all analyses at 5%.

## 3. Results

The mixed ANOVA revealed significant time × group interactions for the mental health (*F*_1,23_ = 6.47; *p* = 0.038; and ηp^2^ = 0.22) and general health (*F*_1,23_ = 15.81; *p* = 0.002; and ηp^2^ = 0.20) dimensions of HRQoL (Table 2). For blood pressure, morphological variables, food consumption frequency, and other dimensions of HRQoL, no significant time × group interactions were found (Table 2). Table 3 shows the results of the time × group interactions of physical fitness, HGS, and postural balance. Regarding physical fitness, significant interactions were revealed in the chair stand (*F*_1,23_ = 6.43; *p* = 0.038; and ηp^2^ = 0.19), arm curl (*F*_1,23_ = 13.55; *p* = 0.007; and ηp^2^ = 0.36), 2-min step (*F*_1,23_ = 6.34; *p* = 0.039; and ηp^2^ = 0.36), and chair sit-and-reach (*F*_1,23_ = 9.07; *p* = 0.019; and ηp^2^ = 0.28) tests. Similarly, significant interactions were only exhibited in postural balance for the eyes-closed condition in the center of the pressure area (*F*_1,23_ = 5.29; *p* = 0.033; and ηp^2^ = 0.07), mean velocity (*F*_1,23_ = 6.19; *p* = 0.041; and ηp^2^ = 0.21), and mediolateral velocity (*F*_1,23_ = 5.66; *p* = 0.040; and ηp^2^ = 0.17). There were no time × group interactions in HGS and other variables of postural balance.

**Table 2:** Time × group interactions of blood pressure, morphological variables, food consumption frequency, and health-related quality of life in independent older women.

**Variable**	**Assessment**	**Group**	**PRE**		**POST**		**Change (%)**	***F-*value**	***p-*value**	****ηp* ^2^***
			**Mean**	**SD**	**Mean**	**SD**				
Blood pressure	Systolic (mm/hg)	TKD (*n* = 14)	121.7	12.2	122.2	7.9	0.4	0.447	0.524	0.02
		MCT (*n* = 11)	133.0	17.2	137.0	14.1	3.0			
	Diastolic (mm/hg)	TKD (*n* = 14)	75.8	8.5	77.2	5.1	1.8	3.480	0.104	0.11
		MCT (*n* = 11)	77.7	9.6	85.3	8.4	9.7			
Morphological variables	Body weight (kg)	TKD (*n* = 14)	72.5	6.8	71.1	5.9	−1.9	2.696	0.144	0.09
		MCT (*n* = 11)	74.9	7.9	76.9	6.3	2.6			
	BMI (kg/m^2^)	TKD (*n* = 14)	30.3	3.0	29.9	2.8	−1.3	4.311	0.076	0.13
		MCT (*n* = 11)	31.4	3.3	32.6	2.7	3.8			
	Fat mass (%)	TKD (*n* = 14)	29.5	6.0	29.2	4.8	−1.0	1.159	0.317	0.05
		MCT (*n* = 11)	32.0	6.8	33.4	4.7	4.3			
	Fat-free mass (%)	TKD (*n* = 14)	23.1	1.4	23.7	1.9	2.6	0.255	0.628	0.01
		MCT (*n* = 11)	22.9	2.5	23.8	1.9	3.9			
Food consumption frequency	Healthy food	TKD (*n* = 14)	37.4	4.8	36.0	11.1	−3.7	0.955	0.361	0.04
		MCT (*n* = 11)	41.8	4.6	43.3	4.9	3.5			
	Unhealthy food	TKD (*n* = 14)	13.3	4.2	10.8	2.5	−18.8	5.298	0.054	0.19
		MCT (*n* = 11)	11.8	2.4	12.7	3.0	7.6			
Health-related quality of life	PF (%)	TKD (*n* = 14)	76.0	16.4	81.44	14.7	7.1	0.083	0.780	0.02
		MCT (*n* = 11)	71.3	12.2	74.5	13.3	4.4			
	BP (%)	TKD (*n* = 14)	67.5	18.9	68.3	21.9	1.1	2.205	0.161	0.07
		MCT (*n* = 11)	67.9	25.6	83.84	13.9	23.4			
	RP (%)	TKD (*n* = 14)	94.6	20.0	100.0	0.0	5.7	0.001	0.999	0.03
		MCT (*n* = 11)	100.0	0.0	100.0	0.0	0.0			
	RE (%)	TKD (*n* = 14)	100.0	0.0	100.0	0.0	0.0	0.001	0.999	0.00
		MCT (*n* = 11)	100.0	0.0	100.0	0.0	0.0			
	MH (%)	TKD (*n* = 14)	56.2	17.3	71.44	16.0	27.1	6.472	0.038^*^	0.22
		MCT (*n* = 11)	65.8	15.6	62.55	6.2	−4.9			
	SF (%)	TKD (*n* = 14)	72.3	27.8	71.4	19.2	−1.2	0.003	0.849	0.00
		MCT (*n* = 11)	75.0	15.8	76.1	14.2	1.4			
	VT (%)	TKD (*n* = 14)	62.5	12.3	71.1	16.1	13.7	0.870	0.381	0.03
		MCT (*n* = 11)	60.9	14.8	62.2	9.5	2.1			
	GH (%)	TKD (*n* = 14)	51.4	20.2	66.73	20.8	29.8	15.810	0.002^**^	0.20
		MCT (*n* = 11)	47.7	16.9	62.77	14.7	31.5			

SD, Standard deviation; TKD, Adapted taekwondo group; MCT, Multicomponent training group; PF, Physical function; RP, Role physical; BP, Bbody pain; VT, Vitality; SF, Social function; RE, Role emotional; MH, Mental health; GH, General health; η*p*^2^, Partial eta squared.

* p < 0.05.

** p < 0.01.

Bold values represent significant differences.

**Table 3:** Time × group interactions of the physical fitness, handgrip strength, and postural balance in independent older women.

**Variable**	**Assessment**	**Group**	**PRE**		**POST**		**Change (%)**	***F-*value**	***p-*value**	****ηp* ^2^***
			**Mean**	**SD**	**Mean**	**SD**				
Physical fitness	Chair stand (Rep)	TKD (*n* = 14)	15.8	4.2	20.1	3.0	27.2	6.436	0.038^*^	0.19
		MCT (*n* = 11)	14.7	3.4	15.3	2.3	4.0			
	Arm curl (Rep)	TKD (*n* = 14)	21.0	5.0	31.7	5.2	50.9	13.550	0.007^**^	0.36
		MCT (*n* = 11)	21.2	4.9	24.2	3.0	14.1			
	2-min step (Rep)	TKD (*n* = 14)	96.1	14.5	115.7	6.9	20.4	6.339	0.039^*^	0.36
		MCT (*n* = 11)	95.0	16.5	99.5	8.7	4.7			
	Chair sit-and-reach (cm)	TKD (*n* = 14)	1.5	5.3	6.0	4.6	300.0	9.072	0.019^*^	0.28
		MCT (*n* = 11)	3.0	6.3	2.9	5.0	−3.3			
	Back scratch (cm)	TKD (*n* = 14)	−6.1	6.4	−2.5	3.8	−59.0	5.418	0.052	0.16
		MCT (*n* = 11)	−4.4	6.3	−5.1	5.2	15.9			
	Timed up-and-go (s)	TKD (*n* = 14)	5.2	0.4	5.1	0.7	−1.9	3.169	0.118	0.12
		MCT (*n* = 11)	5.5	0.9	5.9	0.8	7.2			
Handgrip strenght	Dominant hand (kg)	TKD (*n* = 14)	22.1	3.4	23.0	2.9	4.0	0.002	0.961	0.00
		MCT (*n* = 11)	22.2	3.6	23.1	3.1	4.0			
	Non-dominant hand (kg)	TKD (*n* = 14)	20.6	3.7	22.0	3.1	6.8	0.811	0.397	0.03
		MCT (*n* = 11)	23.0	3.6	23.3	2.3	1.3			
Postural balance	Area EO (cm^2^)	TKD (*n* = 14)	33.8	26.2	14.8	13.4	−56.2	0.949	0.362	0.04
		MCT (*n* = 11)	35.7	23.5	22.7	21.1	−36.4			
	Mean velocity EO (cm/s)	TKD (*n* = 14)	2.5	0.2	2.3	0.1	−8.0	2.328	0.170	0.09
		MCT (*n* = 11)	2.4	0.1	2.3	0.1	−4.2			
	ML velocity EO (cm/s)	TKD (*n* = 14)	3.0	0.6	2.5	0.2	−16.7	0.125	0.724	0.00
		MCT (*n* = 11)	3.6	2.4	2.8	0.8	−22.2			
	AP velocity EO (cm/s)	TKD (*n* = 14)	5.8	1.9	4.2	1.2	−27.6	2.151	0.185	0.09
		MCT (*n* = 11)	5.3	1.1	4.6	1.5	−13.2			
	Area EC (cm^2^)	TKD (*n* = 14)	38.1	26.8	18.6	15.7	−51.2	5.290	0.033^*^	0.07
		MCT (*n* = 11)	37.8	33.0	23.0	20.7	−39.2			
	Mean velocity EC (cm/s)	TKD (*n* = 14)	2.7	0.5	2.4	0.2	−11.1	6.192	0.041^*^	0.21
		MCT (*n* = 11)	2.4	0.1	2.4	0.1	0.0			
	ML velocity EC (cm/s)	TKD (*n* = 14)	2.9	0.4	2.5	0.2	−13.8	5.66	0.040^*^	0.17
		MCT (*n* = 11)	2.7	0.4	2.6	0.3	−3.7			
	AP velocity EC (cm/s)	TKD (*n* = 14)	5.7	1.4	4.8	1.0	−15.8	1.251	0.300	0.05
		MCT (*n* = 11)	5.0	0.9	4.6	0.8	−8.0			

SD, Standard deviation; TKD, Adapted taekwondo group; MCT, Multicomponent training group; EO, Eyes opened; EC, Eyes closed; ML, Mediolateral; AP, Anteroposterior; η*p*^2^, Partial eta squared.

* p < 0.05.

** p < 0.01.

Bold values represent significant differences.

**Figure 3:**
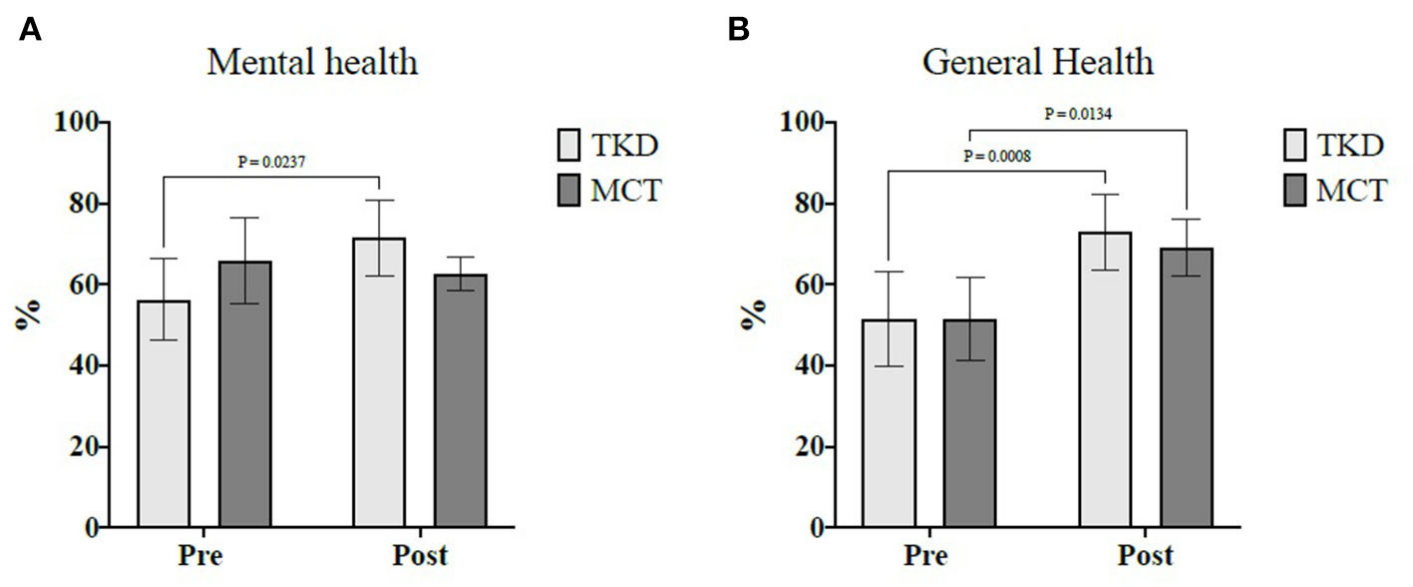
Multiple comparisons of health-related quality of life dimensions showed a significant time × group interaction in independent older women. **(A)** Mental health and **(B)** General health. TKD, Adapted taekwondo group; MCT, Multicomponent training group.

The *post-hoc* analyses showed that, in the TKD, there were significant improvements in the assessments of the mental health (*p* = 0.024; ES = 0.91) and general health (*p* < 0.001; ES = 0.75) dimensions of HRQoL, while in the MCT, there were significant improvements in the general health dimension (*p* = 0.013; ES = 0.95) of HRQoL (Figure 3). Intergroup comparisons showed no significant differences.

**Figure 4:**
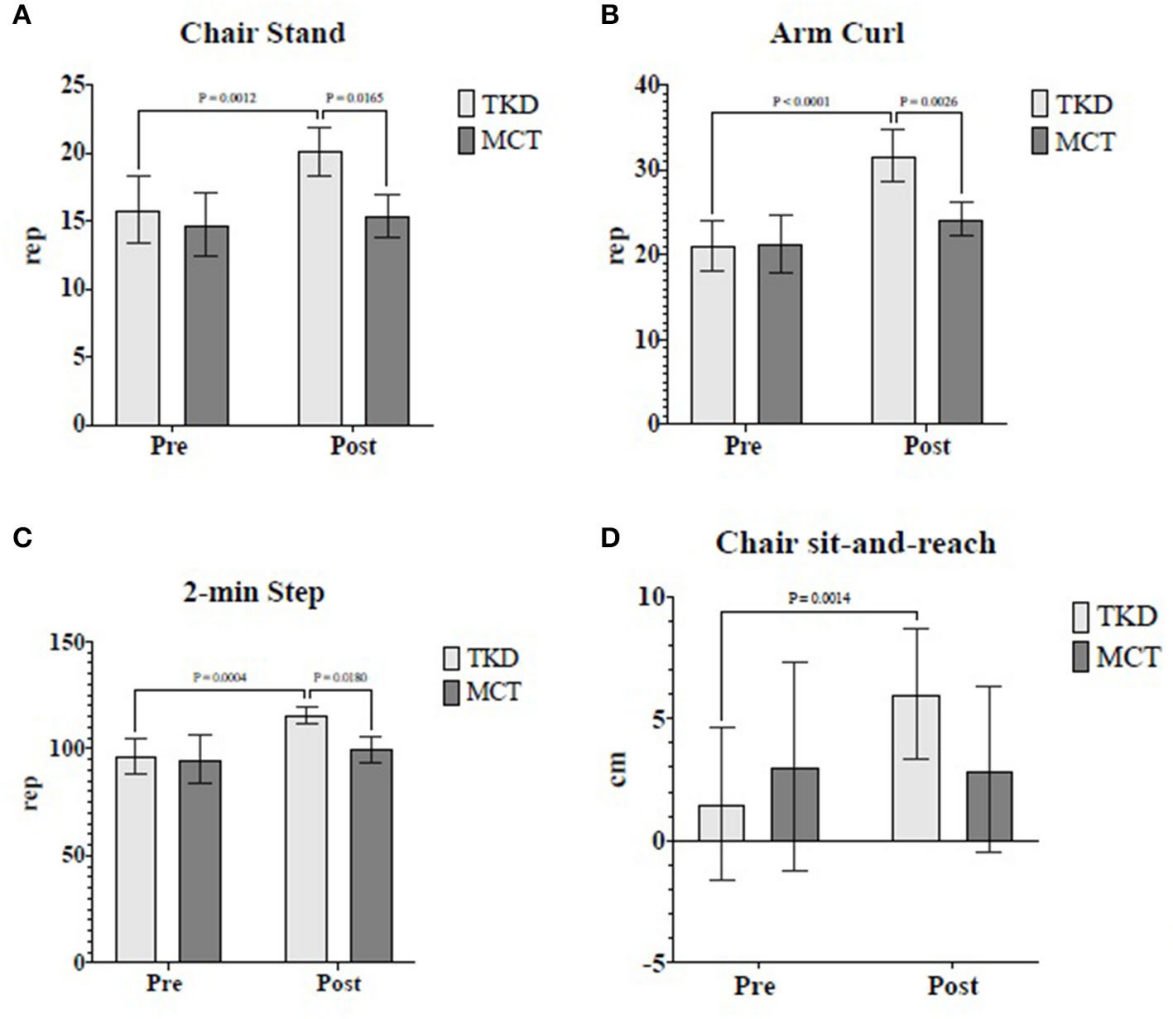
Multiple comparisons of physical fitness variables showed a significant time × group interaction in independent older women. **(A)** Chair stand, **(B)** Arm curl, **(C)** 2-min step, and **(D)** Chair sit-and-reach. TKD, Adapted taekwondo group; MCT, Multicomponent training group.

However, in the TKD, physical fitness tests showed significant improvements in the performance of the chair stand (*p* = 0.001; ES = 1.18), arm curl (*p* < 0.001; ES = 2.10), 2-min step (*p* < 0.001; ES = 1.73), and chair sit-and-reach (*p* = 0.001; ES = 0.91) tests (Figure 4). Multiple comparisons revealed no significant pre- and post-intervention differences in the MCT. For the chair stand (*p* = 0.017; ES = 1.79), arm curl (*p* = 0.003; ES = 1.77), and 2-min step (*p* = 0.018; ES = 0.91) tests, significant differences were observed between the groups in the post-intervention assessment, favoring the TKD group.

**Figure 5:**
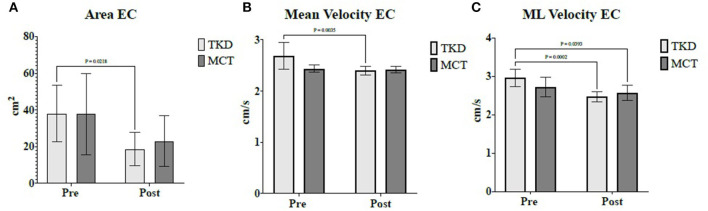
Multiple comparisons of postural balance variables showed a significant time × group interaction in independent older women. **(A)** Area eyes closed, **(B)** Mean velocity eyes closed, and **(C)** Mediolateral velocity eyes closed. TKD, Adapted taekwondo group; MCT, Multicomponent training group; EC, Eyes closed; ML, Mediolateral.

Finally, in the TKD, multiple comparisons revealed a significant reduction in postural balance for the eyes-closed condition in the center of the pressure area (*p* = 0.021; ES = 0.89), mean velocity (*p* = 0.004; ES = 0.79), and mediolateral velocity (*p* < 0.001; ES = 1.26). In the MCT, the mediolateral velocity of postural balance for the eyes-closed condition significantly reduced (*p* = 0.039; ES = 0.28) (Figure 5). Intergroup comparisons showed no significant differences.

The baseline secondary outcomes revealed that, in general, the independent older women analyzed in this study had a mean age of 63.00 ± 2.16 years. Furthermore, 56% of them had a primary academic level, 36% had a secondary academic level, and 8% had a bachelor's or postgraduate degree. Additionally, it can be noted that 76% were married, 16% were separated, and 8% were widowed. Finally, 92% did not smoke (Table 4).

**Table 4:** Baseline assessments of age, academic level, civil status, and smoking status of independent older women.

**Variable**	**Assessment**	**TKD (*n* = 14)**	**MCT (*n* = 11)**	**General sample (*n* = 25)**
Age (years)^*^		62.86 ± 2.38	63.18 ± 1.94	63.00 ± 2.16
Academic level	Primary (%)	28	28	56
	Secondary (%)	24	12	36
	Bachelor (%)	4	0	4
	Postgraduate (%)	0	4	4
Civil status	Married (%)	48	28	76
	Separated (%)	8	8	16
	Widowed (%)	0	8	8
	Single (%)	0	0	0
	Others (%)	0	0	0
Smoking status	Yes (%)	4	4	8
	No (%)	52	40	92

* Data expressed as mean and standard deviation.

TKD, Adapted taekwondo group; MCT, Multicomponent training group.

## 4. Discussion

This study aimed to analyze and compare the effects of an adapted taekwondo program comprising multicomponent training on blood pressure, morphological variables, food consumption frequency, HRQoL, physical fitness, HGS, and postural balance in independent older women. The primary outcomes indicated the following: (*i*) The TKD obtained significantly higher post-intervention results in the chair stand, arm curl and 2-min step tests than the MCT; (*ii*) the TKD significantly increased the mental health and general health dimensions of HRQoL and significantly improved performance in the chair stand, arm curl, 2-min step, and chair sit-and-reach tests of physical fitness, in addition to achieving significant improvements in postural balance for the eyes-closed condition in the center of the pressure area, mean velocity, and mediolateral velocity; (*iii*) the MCT significantly increased the general health dimension of HRQoL and improved postural balance for the eyes-closed condition in mediolateral velocity; and (*iv*) there are no significant changes in the TKD and MCT for blood pressure, morphological variables, food consumption frequency, HGS, back scratch test, and TUG, in six dimensions of HRQoL (physical function, role physical, body pain, vitality, social function, and role emotional) and postural balance for the eyes-open condition after 8 weeks of intervention. Based on our outcomes, the hypothesis was partially confirmed.

Blood pressure, morphological variables, and food consumption frequency did not present post-intervention changes in either the TKD or the MCT. Contrary to this finding, another previously adapted taekwondo intervention reported a significant reduction in body fat percentage, systolic blood pressure, and diastolic blood pressure in older women with depression when compared to an inactive control group (9). It has been reported that older people who participate in physical activity interventions enriched with nutrition education tend to select healthier food options than those who do not have such advice (32). Not finding beneficial changes in the mentioned variables with the TKD and MCT probably indicates the lack of nutritional advice. Nutritional education has been shown to have beneficial effects on food consumption frequency and body composition (33), while reducing the consumption of high-energy-density foods (foods rich in salt, sugar, and fat), such as ultra-processed foods. The reduction in high-energy-density foods' consumption can help reduce systolic and diastolic blood pressure in older people (34). Therefore, an adapted combat sports strategy targeting older people seeking changes in morphological variables and food consumption frequency should incorporate nutritional education. This can generate changes in participants' lifestyles (32, 33), leading to a positive impact on systolic and diastolic blood pressure.

HRQoL did not reveal significant differences between the TKD and MCT. Nonetheless, although the participants presented favorable baseline values for most HRQoL dimensions, they showed improved mental health in the TKD and improved general health in both the TKD and MCT post-intervention. This observation is significant as it has been linked to poor morphological variables and physical fitness; for example, among the HRQoL dimensions, body mass, waist circumference, back scratch test, and TUG were significantly associated with low mental and general health in physically active older women (35). Furthermore, our findings are consistent with interventions based on Olympic combat sports (7) and multicomponent training (16), which have reported beneficial small-to-moderate ES on HRQoL in middle-aged and older people. Older women are a vulnerable group facing the perception of HRQoL because they face more adverse and traumatic life events than older men, along with more frequent negative thoughts and intrusive memories than older men (36). Hence, participating in regular physical activity programs can help promote their general wellbeing (35).

Regarding physical fitness, significant differences were observed in favor of the TKD compared to the MCT in the post-intervention chair stand, arm curl, and 2-min step tests. Furthermore, the TKD demonstrated significant improvements in the results of the chair sit-and-reach test, but no significant changes were observed in the results of the back scratch test, TUG, and HGS. As for the MCT, it did not demonstrate significant changes in the physical fitness tests and HGS. Similar results were presented in previous studies that adapted taekwondo for older women; these studies reported substantial improvements in chair stand (9, 10), arm curl (10), and sit-and-reach (10) tests in favor of the taekwondo group. In addition, they found significant improvements in HGS (9, 11), which we did not detect in our study. However, previous studies (9–11) included control groups that continued their usual activities of daily living, unlike the present study that compared two training programs (TKD vs. MCT). Even though both groups (TKD and MCT) trained at the same intensity (50–70% of the HRmax), the TKD improved the results of the 2-min step test (related to cardiorespiratory fitness). This improvement was probably because of improvements in the chair stand test (related to the muscle strength of lower limbs). The improvements in the chair stand tests may have been due to the specificity of taekwondo's technical foundations, especially the knee raise achieved through hip flexion during kicks (which involve unipodal supports), which could also influence postural balance. In this sense, improving performance in tests related to muscle strength of the upper and lower limbs, cardiorespiratory fitness, and the flexibility of the lower limbs is associated with preventing or reducing sarcopenia (37) and achieving greater autonomy and independence in basic activities of daily living (16), which collectively favor active and healthy aging.

Although there were significant interactions in postural balance for the eyes-closed condition in the center of the pressure area, mean velocity, and mediolateral velocity, no significant differences between the TKD and MCT could be found. Nevertheless, both the TKD and MCT showed a substantial reduction in postural oscillations in the eyes-closed condition in mediolateral velocity. Furthermore, the TKD reduced postural instabilities, which was reflected in the decrease in the center of the pressure area and mean velocity. Similar to this finding, one study reported a significant improvement in postural balance in the eyes-open condition for both the adapted taekwondo and walking exercise groups compared to the passive control group, which was reflected in the decrease in the center of the pressure area, mean velocity, and mediolateral velocity (38). For its part, multicomponent training has been described as the best physical activity strategy to improve, among other variables, the rate of falls and balance performance in physically frail older adults (39). Carrying out interventions that lead to enhanced postural balance in older adults is crucial to decreasing fall risk in healthy older people because, during aging, there is a deterioration in static, dynamic, reactive, or multitasking balance (40), and falls are the second leading cause of death from unintentional injuries worldwide (41).

Some of the possible limitations of the study are as follows: (*i*) The lack of control and follow-up on food consumption, which could influence blood pressure, morphological variables, food consumption frequency, physical fitness, HGS, and postural balance in older women and (*ii*) the lack of an inactive control group to make a complete comparison between the training programs. Some of the main strengths of the study are as follows: (*i*) The comparison of two physically active groups (TKD vs. MCT) and the initial randomization of participants, which increased the study's internal consistency; (*ii*) the use of validated assessments that are widely used in scientific literature, which increased external validity, and (*iii*) the use of training programs tailored to the characteristics of older women, which reduced injury risk and increased adherence to interventions. Future studies could include men and women in the interventions to analyze the possible similarities or differences between the genders, in addition to including an inactive control group, which would help analyze the physical activity programs.

Finally, despite the statistical differences observed between the two groups (TKD and MCT) concerning the variables analyzed, the more effective intervention method for promoting health among older women may be related to intrinsic choices and participants' adherence to maintaining their participation in the intervention (42). Thus, in health promotion actions undertaken as part of municipal, state, or national interventions, it may be more advantageous to offer participants the freedom to choose the type of physical activity programs that best align with their preferences, thereby promoting increased adherence, satisfaction, and pleasure. Consequently, positive outcomes in terms of physical, nutritional, and psychological status can be expected, highlighting the importance of the principle of continuity of training for older people.

## 5. Conclusion

The multiple comparisons showed that the adapted taekwondo program achieved significantly higher results in the chair stand, arm curl, and 2-min step tests than multicomponent training in independent older women. In addition, the TKD offered beneficial and significant changes in mental health, the general health dimension of HRQoL, the chair sit-and-reach test, and postural balance for the eyes-closed condition, specifically in the area, mean velocity, and mediolateral velocity. In contrast, the MCT showed significant improvements in the general health dimension of HRQoL and postural balance for the eyes-closed condition, specifically, in mediolateral velocity. Compared to multicomponent training, taekwondo improves postural balance and achieves better benefits at physical fitness and HRQoL levels among older women. Therefore, it is possible to recommend taekwondo as a safe physical activity strategy, given its high adherence to intervention among older women when following the dosage and activities proposed in this study's program.

## Data availability statement

The original contributions presented in the study are included in the article/supplementary material, further inquiries can be directed to the corresponding author.

## Ethics statement

The studies involving humans were approved by the Scientific Ethics Committee of the Universidad Católica del Maule, Chile (Number: N°29-2022). The studies were conducted in accordance with the local legislation and institutional requirements. The participants provided their written informed consent to participate in this study.

## Author contributions

PV-B was responsible for conceptualizing the study. The methodology was developed by PV-B, EG-M, and TH-V. PV-B and EG-M were involved in the implementation of software. Formal analysis was conducted by PV-B, EG-M, and TH-V. The investigation involved the efforts of PV-B, EG-M, TH-V, BB, JH-M, and HN. PV-B prepared the initial draft and review and editing were performed by PV-B, EG-M, TH-V, BB, JH-M, and HN. PV-B, EG-M, and TH-V supervised the project. All authors have reviewed and approved the final version of the manuscript for publication.
